# TA-TMA as a clinical model for precision complement therapeutics in stem cell transplantation

**DOI:** 10.3389/fimmu.2026.1959683

**Published:** 2026-10-12

**Authors:** Sonata Jodele

**Affiliations:** 1 Division of Bone Marrow Transplantation and Immune Deficiency, Cancer and Blood Disease Institute, Cincinnati Children’s Hospital Medical Center, Cincinnati, OH, United States; 2 Department of Pediatrics, University of Cincinnati College of Medicine, Cincinnati, OH, United States

**Keywords:** biomarkers, complement, eculizumab, endothelial injury, hematopoietic stem cell transplantation, precision medicine, TA-TMA, transplant-associated thrombotic microangiopathy

## Abstract

Transplant-associated thrombotic microangiopathy (TA-TMA) has evolved from a poorly understood and frequently fatal complication of hematopoietic stem cell transplantation (HSCT) into the leading clinical model for precision complement therapeutics. Studies of TA-TMA have established endothelial injury and complement dysregulation as clinically actionable mechanisms and demonstrated that successful complement inhibition requires more than selecting an effective therapeutic agent. Longitudinal pharmacokinetic/pharmacodynamic (PK/PD)-guided dosing, durable terminal complement blockade, and integration of biomarkers reflecting complement activation, endothelial injury, inflammation, and organ dysfunction have transformed eculizumab into the first example of individualized complement-directed therapy in transplantation. Beyond improving outcomes in patients with TA-TMA, these advances have fundamentally changed our understanding of post-transplant endothelial injury, revealing shared biological pathways across multiple transplant-associated complications and supporting a transition from syndrome-based to mechanism-based clinical management. The expanding availability of eculizumab biosimilars and complement pathway-specific inhibitors, including lectin pathway inhibition, further broadens opportunities for personalized therapeutic strategies while emphasizing the need for harmonized diagnostic criteria, standardized response assessment, and biomarker-guided patient selection. Together, the lessons learned from TA-TMA provide a framework for future precision complement therapeutics in transplantation and illustrate how mechanistic understanding of disease biology can transform clinical practice.

## 1 Introduction

The complement system has emerged as a central mediator of transplant-associated endothelial injury and a promising therapeutic target across hematopoietic stem cell transplantation (HSCT). While complement activation has been implicated in numerous transplant complications, transplant-associated thrombotic microangiopathy (TA-TMA) has become the clearest clinical example in which mechanistic understanding of complement biology has been successfully translated into targeted therapy. Over the past two decades, advances in the diagnosis and management of TA-TMA have transformed a frequently fatal complication into a treatable condition, establishing a paradigm for precision complement therapeutics (**Figure 1**).

**Figure 1:**
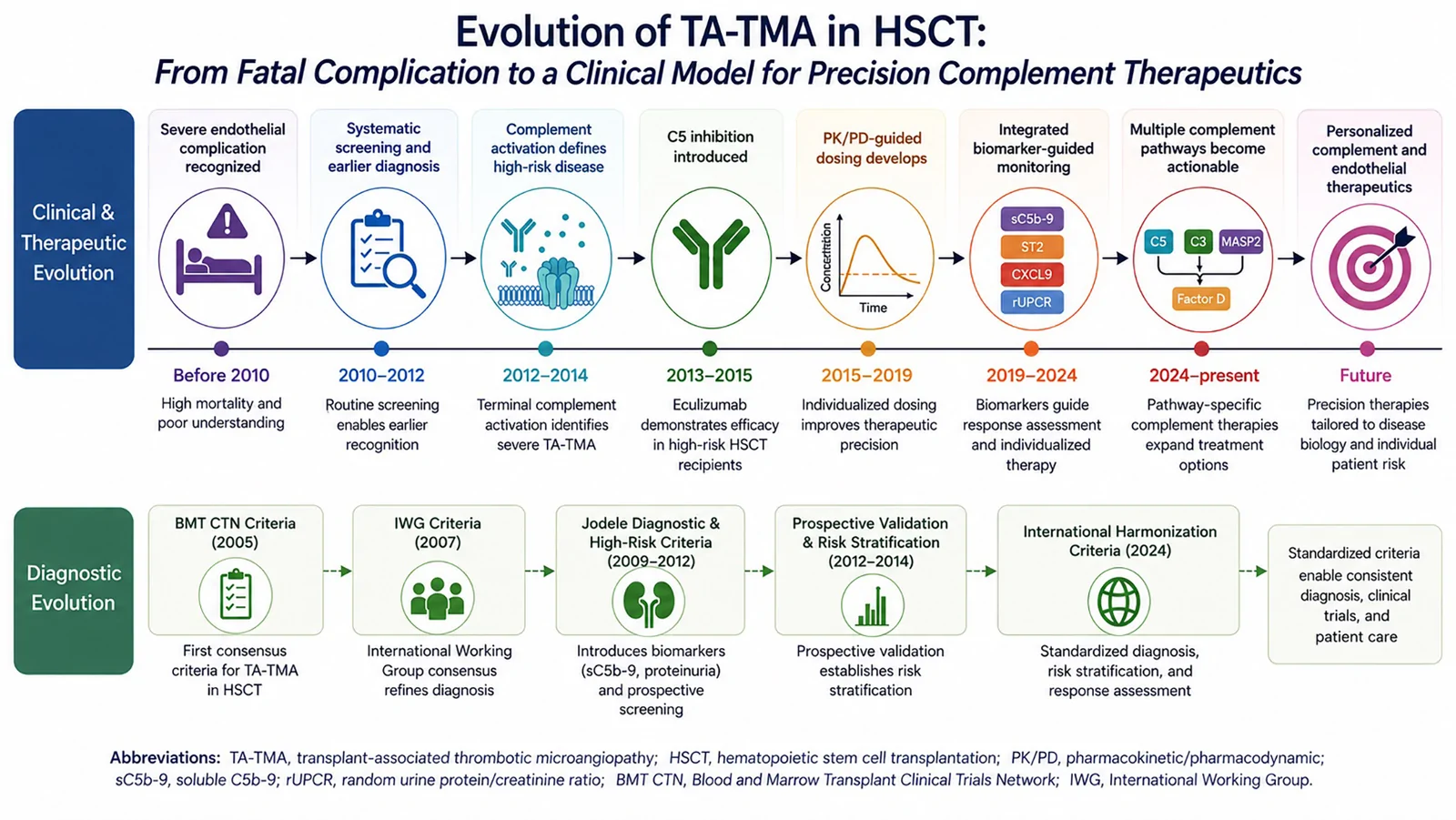
Evolution of TA-TMA in HSCT: from fatal complication to a clinical model for precision complement therapeutics. Timeline highlighting major advances in TA-TMA recognition, diagnostic criteria, complement-directed treatment, and the evolution toward individualized precision therapeutics.

Beyond improving outcomes in patients with TA-TMA, this experience has substantially advanced our understanding of endothelial injury following hematopoietic stem cell transplantation. TA-TMA has demonstrated that endothelial dysfunction is not a static process but rather a dynamic interplay between tissue injury, innate immune activation, inflammation, coagulation, and complement dysregulation. Importantly, these biological pathways are increasingly recognized to extend beyond TA-TMA and contribute to the pathogenesis and severity of other transplant-associated complications, suggesting that lessons learned from TA-TMA may have broader implications for transplantation biology.

Perhaps the most important lesson from the TA-TMA experience is that successful complement inhibition requires more than selecting an effective therapeutic agent. Clinical outcomes depend on timely recognition of complement-mediated disease, individualized pharmacokinetic and pharmacodynamic monitoring to achieve sustained complement blockade, and integration of biomarkers that reflect endothelial injury and inflammatory activity. These principles have transformed eculizumab from an empirically administered drug into the first example of precision complement therapeutics in transplantation.

In this review, we discuss how TA-TMA has served as a clinical model for advancing precision complement therapeutics in transplantation. We summarize the evolution of eculizumab therapy and the lessons learned from individualized dosing and biomarker-guided management, discuss how these advances have expanded our understanding of transplant-associated endothelial injury, review the emerging role of eculizumab biosimilars in improving access to complement-directed therapy, and highlight future opportunities for personalized therapeutic strategies, including integration of complement biology with inflammatory pathways such as the interferon-complement axis.

## 2 TA-TMA: a clinical model for understanding complement-mediated endothelial injury

The recognition of transplant-associated thrombotic microangiopathy (TA-TMA) as a complement-mediated endothelial disorder has fundamentally changed the understanding of post-transplant vascular complications (1, 2). Initially regarded primarily as a clinicopathologic syndrome characterized by microangiopathic hemolytic anemia, thrombocytopenia, and organ dysfunction, TA-TMA is now recognized as a dynamic disease process driven by endothelial injury with secondary activation and amplification of the complement cascade (3, 4). This evolving understanding has provided the biological rationale for targeted complement inhibition and established TA-TMA as a clinical model for precision complement therapeutics in children undergoing HSCT (5–7).

The pathogenesis of TA-TMA is multifactorial. Endothelial injury begins with conditioning-related toxicity and may further amplified by additional post-transplant insults like graft-versus-host disease (GVHD), infections, inflammatory cytokines, and other transplant-associated stressors (8–11). Rather than acting independently, these insults converge on a common pathway of endothelial activation characterized by loss of vascular homeostasis, increased permeability, leukocyte adhesion, platelet activation, and microvascular thrombosis. Within this complex network, complement activation functions as a critical amplifier of endothelial injury, perpetuating inflammation and microvascular dysfunction through a self-sustaining cycle of endothelial damage and complement activation. To facilitate clinical implementation, the current international harmonization diagnostic criteria, high-risk features, and recommended screening strategy are summarized in **Table 1**.

**Table 1:** International consensus diagnostic and risk stratification criteria for TA-TMA.

Clinical step	Current consensus criteria	Practical interpretation
Clinical diagnosis	≥4 of 7 features present at 2 time points within 14 days	No single feature is required. Organ dysfunction is not required for diagnosis.
Anemia	Failure to achieve RBC transfusion independence; OR Hb decline ≥1 g/dL; OR new RBC transfusion dependence	Consider alternative causes after HCT.
Thrombocytopenia	Failure to achieve platelet engraftment; platelet transfusion refractoriness; OR ≥50% platelet decline after engraftment	Interpret in context of other post-HCT causes.
Elevated LDH	Above ULN for age	Supports endothelial injury but is not specific.
Schistocytes	Present on peripheral smear	Supportive but not mandatory.
Hypertension	<18 yr: BP >99th percentile; ≥18 yr: BP ≥140/90 mmHg	Refractory/disproportionate hypertension increases suspicion.
Elevated sC5b-9	Above ULN	Marker of terminal complement activation.
Proteinuria	rUPCR ≥1 mg/mg	Preferred over dipstick proteinuria.
Histologic diagnosis	Renal or intestinal biopsy demonstrating TA-TMA	Biopsy is sufficient but not required.
High-risk TA-TMA	Any: elevated sC5b-9; LDH ≥2×ULN; rUPCR ≥1 mg/mg; organ dysfunction; grade II-IV acute GVHD; bacterial/viral infection	Any feature defines high-risk disease.
Screening	Weekly through day +100 after allogeneic HCT and pediatric autologous neuroblastoma HCT; continue with GVHD/infection	Prospective surveillance enables earlier diagnosis.

TA-TMA is diagnosed clinically using the modified Jodele criteria when ≥4 of 7 features occur at two time points within 14 days. Organ dysfunction is not required for diagnosis and instead contributes to risk stratification.

BP, blood pressure; GVHD, graft-versus-host disease; HCT, hematopoietic cell transplantation; LDH, lactate dehydrogenase; RBC, red blood cell; rUPCR, random urine protein-to-creatinine ratio; sC5b-9, soluble C5b-9; TA-TMA, transplant-associated thrombotic microangiopathy; ULN, upper limit of normal.

One of the most important lessons from TA-TMA is that complement activation exists on a biological continuum rather than as an isolated event. Complement dysregulation varies between patients according to genetic susceptibility, the magnitude and persistence of endothelial injury, and the inflammatory milieu following transplantation (12–17). Consequently, TA-TMA represents not merely excessive complement activation but a failure of physiologic mechanisms that normally restore endothelial homeostasis after transplant-associated injury. This concept helps explain the marked heterogeneity in disease severity, organ involvement, and therapeutic response observed among patients.

Prospective adult studies have further demonstrated that TA-TMA is a clinically important complication across age groups. In the multicenter MIDAS Consortium study (Microangiopathy, endothelial Damage in Adults undergoing Stem cell transplantation), systematic surveillance identified severe TA-TMA in a substantial proportion of adult allogeneic HSCT recipients and confirmed its association with increased nonrelapse mortality and inferior overall survival (18). These findings extend observations from prospective pediatric cohorts while also emphasizing the need to validate diagnostic, prognostic, and therapeutic biomarkers independently in adult populations, where disease biology, transplant exposures, and biomarker performance may differ.

The TA-TMA experience has also broadened our understanding of endothelial injury beyond a single disease entity. The same biological processes that initiate TA-TMA, including endothelial activation, inflammatory signaling, and complement amplification, are increasingly recognized to contribute to other transplant-associated complications, including severe gastrointestinal GVHD, diffuse alveolar hemorrhage, and other manifestations of endothelial dysfunction. While the relative contribution of complement likely differs across these conditions and age groups, TA-TMA has provided an invaluable clinical model for understanding how endothelial injury evolves and how complement-directed interventions may modify disease progression (19–23).

Collectively, these observations established the scientific foundation for precision complement therapeutics. Rather than viewing complement inhibition as a universal treatment for endothelial injury, the TA-TMA experience demonstrated the importance of identifying patients in whom complement activation is a dominant driver of disease, monitoring the adequacy of complement blockade throughout therapy, and integrating biomarkers that reflect endothelial injury and inflammatory activity. These principles have subsequently shaped the development of individualized therapeutic strategies discussed in the following sections.

Although TA-TMA remains primarily a clinical diagnosis and tissue biopsy is not required, histologic confirmation can be particularly valuable in selected cases with organ-predominant disease or when the differential diagnosis is challenging. Current international harmonization criteria recognize kidney and intestinal biopsy demonstrating TA-TMA as sufficient evidence to establish the diagnosis, although TA-TMA has also been documents in other organs. Importantly, when kidney or gastrointestinal tissue is obtained for other clinical indications, including evaluation of graft-versus-host disease, pathologists should routinely assess for histologic features of TA-TMA because endothelial injury frequently coexists with other transplant complications. Histologic confirmation establishes the diagnosis regardless of circulating biomarkers, identifies patients as having high-risk TA-TMA, and supports prompt initiation of complement-directed therapy.

## 3 Eculizumab and the evolution of precision complement therapeutics

The introduction of eculizumab, a humanized monoclonal antibody targeting complement component C5, represented a major milestone in the management of TA-TMA and provided the first successful example of targeted complement inhibition in hematopoietic stem cell transplantation. Beyond improving survival, clinical experience with eculizumab fundamentally changed our understanding of how complement-directed therapies should be administered, monitored, and individualized in patients with severe endothelial injury. Clinical outcomes with eculizumab have varied across published cohorts and treatment settings, likely reflecting differences in disease severity and timing of therapy as well as access to PK/PD monitoring and the ability to optimize drug exposure and achieve sustained complement blockade. Over the past two decades, the field has evolved from empiric complement blockade to precision therapeutic strategies guided by pharmacokinetic (PK), pharmacodynamic (PD), and biomarker monitoring. These lessons extend beyond TA-TMA and provide a framework for the future development of personalized complement therapeutics across transplantation (13, 24–28).

### 3.1 From complement biology to targeted therapy

The recognition that uncontrolled terminal complement activation contributes to endothelial injury in TA-TMA provided the biological rationale for therapeutic complement inhibition. Early clinical observations demonstrated markedly elevated markers of terminal complement activation as measured by plasma soluble C5b-9 (sC5b-9) in patients with severe disease, supporting the hypothesis that complement was not merely a consequence of endothelial injury but an active contributor to ongoing microvascular damage (14, 23, 29–32). These findings led to the off-label use of eculizumab to interrupt terminal complement activation and prevent formation of the membrane attack complex.

Initial clinical experience demonstrated that eculizumab could rapidly halt complement-mediated vascular injury and substantially improve survival in patients with high-risk TA-TMA. These observations established proof-of-concept that targeted complement inhibition could successfully modify the natural history of this previously devastating complication. However, early experience also revealed considerable heterogeneity in treatment response, highlighting that complement inhibition alone was insufficient unless adequate and sustained terminal complement blockade could be achieved (26, 33, 34).

The TA-TMA experience therefore shifted the focus from simply administering a complement inhibitor to understanding the biological determinants of treatment response. This transition laid the foundation for individualized complement therapeutics and ultimately transformed eculizumab from an empiric rescue therapy into a precision treatment strategy.

### 3.2 Why individualized dosing became necessary

Unlike atypical hemolytic uremic syndrome and paroxysmal nocturnal hemoglobinuria, patients with TA-TMA experience profound systemic inflammation, extensive endothelial injury, and ongoing complement dysregulation following HSCT. These disease-specific characteristics produce substantial pharmacokinetic variability, resulting in accelerated eculizumab clearance and highly variable drug exposure among individual patients (34).

Clinical experience soon demonstrated that conventional weight-based dosing schedules frequently failed to maintain continuous terminal complement blockade during periods of intense disease activity. Inadequate drug exposure was associated with incomplete complement inhibition, persistent endothelial injury, and inferior clinical outcomes, emphasizing that standard dosing algorithms developed for other complement-mediated disorders could not simply be extrapolated to TA-TMA.

These observations established one of the most important principles in complement therapeutics: dosing should be guided by the biological activity of the disease rather than by treatment schedules established in other diseases. Individualized dosing based on complement activity and drug pharmacokinetics became essential to achieve consistent target engagement throughout therapy, particularly during the initial phase when complement activation is most pronounced (33).

### 3.3 Eculizumab PK/PD monitoring transformed clinical practice

One of the major advances in complement therapeutics for TA-TMA has been the recognition that effective terminal complement blockade requires adequate drug exposure, pharmacodynamic suppression of complement activity, and longitudinal monitoring of disease biology. Pharmacokinetic (PK) and pharmacodynamic (PD) studies established the biological framework for understanding complement blockade in TA-TMA and demonstrated how biomarker-guided dose adjustment can optimize treatment during active disease. These studies also enabled the development of standardized phase- and weight-based dosing algorithms derived from prospective PK/PD investigations, providing a practical approach for centers where real-time PK/PD monitoring is not readily available. Importantly, these standardized dosing algorithms are distinct from the fixed dosing regimen described in the eculizumab product label for atypical hemolytic uremic syndrome (aHUS), as they were specifically developed and validated for the unique PK characteristics and complement activation observed in high-risk TA-TMA.

Effective complement blockade can therefore be achieved using two complementary approaches: individualized real-time PK/PD-guided therapy (**Figure 2**) or standardized phase- and weight-based dosing algorithms derived from prospective PK/PD studies (Jodele/Mizuno et al, JTCT 2026 in press). In centers with access to real-time monitoring, eculizumab trough concentrations, CH50, and sC5b-9 provide complementary information regarding drug exposure, terminal complement blockade, and systemic complement activation, allowing individualized dose adjustment throughout therapy. Where these biomarkers are unavailable, standardized dosing algorithms derived from prospective PK/PD studies provide a practical alternative for achieving the same biological goals.

**Figure 2:**
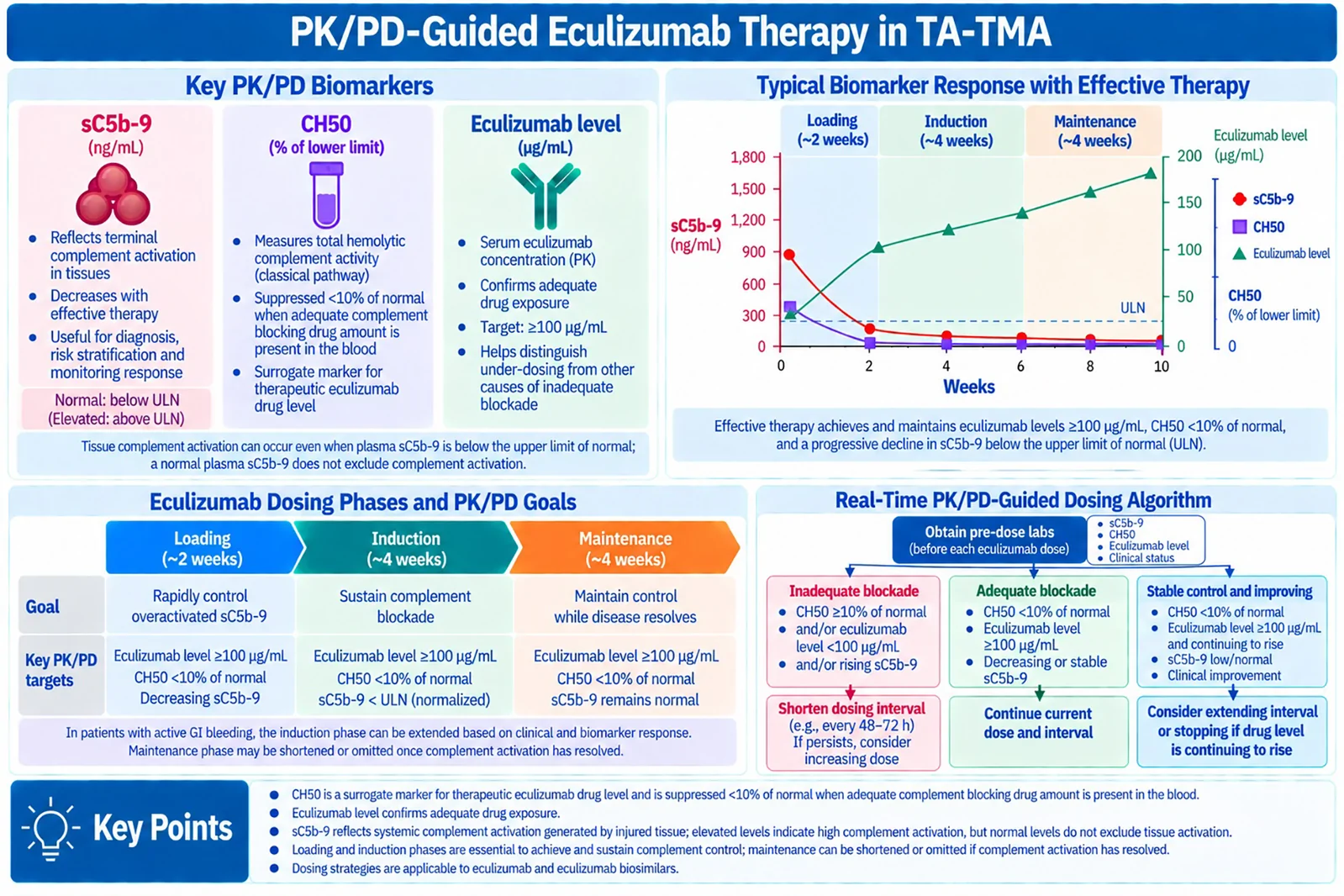
PK/PD-guided eculizumab therapy in TA-TMA. Overview of key pharmacokinetic/pharmacodynamic biomarkers, expected biomarker trajectories during effective complement blockade, treatment phases, and a practical algorithm for individualized eculizumab dosing.

Pharmacokinetic monitoring is based on eculizumab trough concentrations, which directly quantify circulating drug exposure. Pharmacodynamic monitoring uses CH50, a functional assay of total classical pathway hemolytic activity that serves as a practical surrogate marker for adequate circulating complement-blocking drug levels. When sufficient eculizumab is present in the circulation, CH50 remains suppressed to <10% of normal, confirming effective terminal complement blockade within the bloodstream. Recovery of CH50 to at least lower level of normal indicates eculizumab drug clearance from the blood circulation and re-established on complement activity in the blood. In contrast, soluble C5b-9 (sC5b-9) reflects systemic complement activation generated by injured tissues and therefore provides complementary information regarding ongoing disease activity. Progressive decline and eventual normalization of plasma sC5b-9 indicate successful control of systemic complement activation; however, normal plasma sC5b-9 does not exclude ongoing tissue complement activation and should always be interpreted in the context of the patient’s overall clinical status.

The therapeutic objective differs across treatment phases. During the loading phase, adequate circulating drug exposure and rapid suppression of CH50 are achieved while initiating reduction of systemic complement activation with the goal to normalize sC5b-9 in less than two weeks to avoid or prevent tissue injury. During induction, sustained therapeutic drug concentrations and continued CH50 suppression maintain complete terminal complement blockade (normal sC5b-9). Once durable complement control has been established, the maintenance phase maintains disease control while drug clearance decreases (drug levels continue to rise) and complement activation resolves, allowing gradual extension of dosing intervals or discontinuation of therapy when appropriate. Sustaining normal sC5b-9 after eculizumab is discontinued and CH50 normalizes(drug is cleared) indicates successful achievement of complement normalization after therapy.

Failure to achieve adequate PK or PD targets provides practical guidance for treatment adjustment. Detectable CH50, subtherapeutic eculizumab concentrations, or persistently elevated sC5b-9 indicate inadequate complement blockade and generally require shortening the dosing interval before increasing the dose. Conversely, persistently therapeutic drug concentrations with continued CH50 suppression and stable or normalized sC5b-9 support continuation of the current regimen and, during maintenance, gradual extension of dosing intervals or discontinuation once disease activity has resolved. These PK/PD principles are equally applicable to originator eculizumab and currently available eculizumab biosimilars.

**Figure 2** displays practical implementation of PK/PD-guided eculizumab therapy in TA-TMA (5, 34). Together, these parameters permit real-time assessment of treatment adequacy and facilitate individualized dose adjustment throughout therapy.

Importantly, PK/PD-guided therapy demonstrated that successful complement inhibition is a dynamic process requiring repeated reassessment as endothelial injury resolves and complement activation declines. This individualized approach improved the consistency of complement blockade, shortened unnecessary treatment in many patients, and established precision therapeutic monitoring as a cornerstone of TA-TMA management (24).

### 3.4 Durable complement blockade precedes clinical recovery

One of the most important biological insights gained from longitudinal monitoring is that pharmacodynamic complement control precedes clinical recovery. Terminal complement blockade can often be achieved within weeks of therapy initiation, whereas normalization of hematologic parameters, renal function, gastrointestinal injury, and other manifestations of endothelial recovery frequently occur substantially later.

Recent multicenter real-world data further investigated the expected timeline of clinical response. In a seven-center pediatric cohort evaluated using international consensus response definitions, failure to achieve at least a partial response by day 45 was associated with a low likelihood of subsequent response and substantially increased mortality. These findings support approximately six weeks after therapy initiation as a clinically meaningful time point for comprehensive reassessment, including confirmation of adequate complement blockade, evaluation of biomarker trajectories and organ recovery, and consideration of alternative or additional therapeutic strategies when no biological or clinical response is evident (28).

This temporal dissociation highlights the distinction between suppression of disease activity and restoration of organ function. Complement inhibition interrupts ongoing endothelial injury, but recovery requires subsequent repair of damaged microvasculature and resolution of downstream inflammatory and thrombotic consequences. Consequently, assessment of therapeutic success should not rely solely on immediate clinical improvement but should incorporate objective measures confirming sustained complement blockade.

These observations have important implications for clinical decision-making. Failure to recognize the delayed nature of endothelial recovery may lead to premature modification or discontinuation of effective therapy, whereas persistent complement activation despite treatment identifies patients who require continued therapeutic intensification or consideration of alternative treatment strategies.

Recent international consensus recommendations have also harmonized response assessment for TA-TMA-directed therapy by defining complete response, clinically meaningful partial response, and no response using integrated hematologic, complement, and organ-specific criteria. These definitions acknowledge that biological response often precedes complete organ recovery and provide a standardized framework for evaluating treatment efficacy in both clinical practice and future clinical trials. A practical summary is provided in **Table 2**.

**Table 2:** International consensus response criteria for TA-TMA–directed therapy.

Response domain	Complete response (CR)	Clinically meaningful partial response (PR)	No response (NR)
Overall hematologic/ biochemical response	Resolution of RBC and platelet transfusion dependence; normalization of sC5b-9; and CR of either LDH or schistocytes.	Resolution of RBC and platelet transfusion dependence; ≥25% reduction in sC5b-9 (if elevated); and PR of either LDH or schistocytes.	Persistent transfusion dependence or no meaningful improvement in hematologic/biochemical markers.
Anemia	No RBC transfusions for 14 days and hemoglobin normal for age.	No RBC transfusions for 7 days or clinically meaningful hemoglobin improvement.	Persistent transfusion dependence or no improvement.
Thrombocytopenia	No platelet transfusions for 14 days and platelet recovery.	No platelet transfusions for 7 days with platelet recovery.	Persistent transfusion dependence or no improvement.
LDH/Schistocytes	LDH normalized or schistocytes resolved.	≥25% LDH reduction or reduction in schistocytes.	No meaningful improvement.
sC5b-9	Normal (<ULN) at two assessments within 14 days.	≥25% reduction from diagnosis if still above ULN.	No meaningful improvement.
Overall organ response	All involved organs meet CR criteria.	All involved organs meet at least PR criteria.	Failure of any involved organ to achieve PR.
Practical organ assessment	Return to pre-HSCT or age-normal function; no organ-support therapy for 14 days when applicable.	Objective improvement in organ function; no organ-support therapy for 7 days when applicable.	Persistent organ dysfunction/failure.
Overall TA-TMA response	CR of all hematologic/biochemical and organ manifestations.	At least PR in all hematologic/biochemical and organ manifestations (mixed CR/PR = overall PR).	Any hematologic/biochemical NR or failure of any organ.

Response assessment should evaluate hematologic/biochemical and organ manifestations separately, followed by an overall response assessment. Hematologic improvement often precedes organ recovery.

CR, complete response; PR, clinically meaningful partial response; NR, no response; HCT, hematopoietic cell transplantation; LDH, lactate dehydrogenase; RBC, red blood cell; sC5b-9, soluble C5b-9; TA-TMA, transplant-associated thrombotic microangiopathy; ULN, upper limit of normal.

Organ-specific response criteria (renal, proteinuria, hypertension, pulmonary, gastrointestinal, serositis, and CNS manifestations) are summarized from the International Consensus Response Criteria. This condensed table is intended as a practical clinical reference.

### 3.5 Principles of precision complement therapeutics

The cumulative experience with eculizumab has extended far beyond the treatment of TA-TMA and has established several fundamental principles for precision complement therapeutics. First, successful therapy depends on identifying patients in whom complement activation is a major driver of disease rather than assuming uniform benefit across all forms of endothelial injury. Second, therapeutic success requires confirmation of sustained pharmacodynamic target engagement through longitudinal PK/PD monitoring rather than reliance on fixed dosing schedules. Third, biomarkers reflecting complement activation, endothelial injury, and inflammatory activity provide complementary information that improves assessment of treatment response and disease evolution (14, 23, 29, 30).

Collectively, these lessons have transformed eculizumab from a targeted biologic into a model for precision medicine in transplantation. As biosimilars and next-generation complement inhibitors become increasingly available, these principles are likely to remain broadly applicable irrespective of the specific therapeutic agent used. The future of complement therapeutics will therefore depend not only on developing new inhibitors but also on integrating mechanistic understanding, individualized monitoring, and biomarker-guided clinical decision-making into routine transplant care.

## 4 Biomarkers for precision therapeutics

Although durable terminal complement blockade is essential for successful therapy, complement inhibition alone does not fully capture the biological complexity of TA-TMA. Longitudinal clinical observations have demonstrated that patients with similar pharmacodynamic complement control may experience markedly different rates of endothelial recovery and clinical improvement. These findings suggest that complement activity represents only one component of disease biology and that additional biomarkers reflecting endothelial injury, inflammation, and organ dysfunction are required to more comprehensively assess therapeutic response. The integration of complementary and endothelial injury biomarkers has therefore become an important component of precision therapeutic monitoring in TA-TMA (35–40).

### 4.1 Soluble C5b-9: monitoring systemic complement activation

Soluble C5b-9 (sC5b-9), a marker of terminal complement activation, has played a central role in advancing the diagnosis and management of TA-TMA. Initially incorporated as a biomarker to identify complement-mediated disease, its clinical utility has expanded considerably through longitudinal clinical experience. Today, sC5b-9 serves multiple complementary roles, including supporting the diagnosis of TA-TMA, identifying patients with high-risk disease, monitoring response to complement inhibition, and guiding individualized therapeutic decision-making.

Perhaps the most important lesson learned is that sC5b-9 reflects systemic terminal complement activation, providing biological evidence that complement dysregulation extends beyond isolated organ injury and contributes to widespread endothelial dysfunction. Elevated sC5b-9 levels have consistently been associated with more severe disease, greater risk of multi-organ involvement, and inferior clinical outcomes, supporting its role as both a diagnostic and prognostic biomarker.

Longitudinal monitoring in children undergoing HSCT has further demonstrated that interpretation of sC5b-9 should extend beyond absolute laboratory thresholds. While population-based reference ranges remain clinically useful, individual patients often have their own biological baseline pre-transplant. Progressive elevation from a patient’s pre-transplant baseline may therefore signal evolving complement activation before reaching predefined diagnostic cutoffs, emphasizing the importance of serial measurements and trend analysis rather than isolated values. Likewise, declining sC5b-9 concentrations during therapy provide evidence of biological response (23).

Collectively, the TA-TMA experience has transformed sC5b-9 from a diagnostic laboratory marker into a dynamic biomarker of disease biology in pediatric HSCT. When interpreted longitudinally and integrated with PK/PD monitoring and complementary markers of endothelial injury and inflammation, sC5b-9 provides critical insight into disease severity, therapeutic response, and the evolving biological state of the patient.

While sC5b-9 has emerged as the most established biomarker of terminal complement activation in pediatric TA-TMA and has proven valuable for diagnosis, risk stratification, and therapeutic monitoring, emerging prospective adult studies suggest that biomarker performance may differ across age groups and transplant populations. These observations highlight the importance of validating biomarkers within specific clinical settings and emphasize that precision medicine requires understanding not only what a biomarker measures biologically, but also in whom and when it is most informative (18, 38, 40).

### 4.2 Endothelial injury and organ recovery biomarkers

While complement activation is central to TA-TMA pathogenesis, longitudinal studies have demonstrated that biomarkers reflecting endothelial injury and organ dysfunction provide complementary information regarding disease activity and therapeutic response. These biomarkers help distinguish successful complement inhibition from ongoing endothelial injury and tissue recovery, reinforcing that restoration of vascular homeostasis extends beyond normalization of complement activity (**Figure 3**).

**Figure 3:**
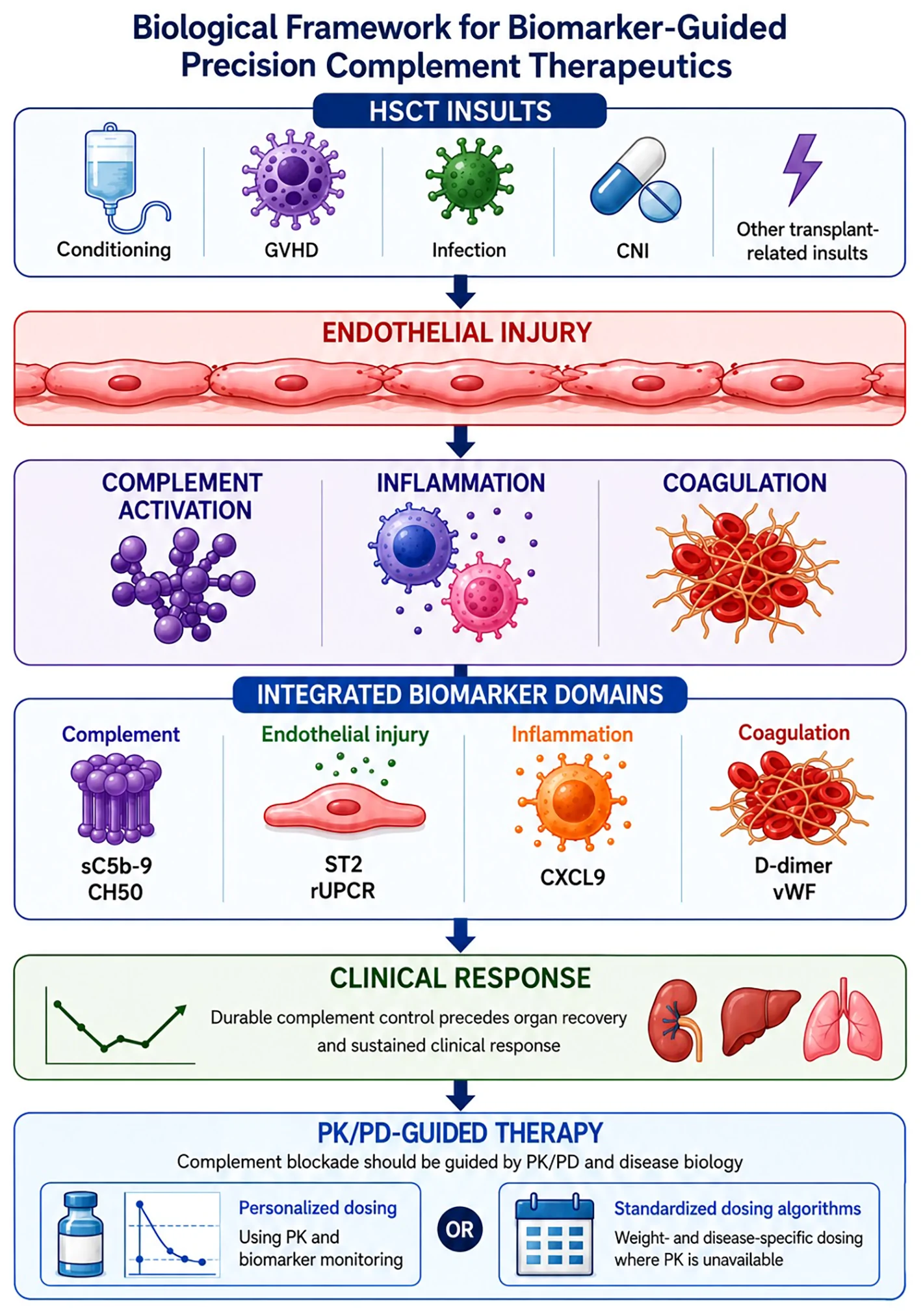
Biological framework for biomarker-guided precision complement therapeutics. Schematic illustrating how transplant-associated insults initiate endothelial injury and activate complement, inflammatory, and coagulation pathways. Integration of biomarkers across these biological domains supports therapeutic monitoring and assessment of clinical recovery.

Among circulating biomarkers, soluble suppression of tumorigenicity-2 (ST2) has emerged as an important indicator of endothelial stress and vascular injury (35, 41–43). Although ST2 is widely incorporated into the Mount Sinai Acute GVHD International Consortium (MAGIC) algorithm as a prognostic biomarker for acute graft-versus-host disease (GVHD), it is important to recognize that ST2 is not specific for GVHD (44, 45). Increasing evidence demonstrates that ST2 reflects endothelial injury and systemic vascular inflammation and is consistently elevated in patients with TA-TMA. Consequently, elevated ST2 should be interpreted in the broader context of transplant-associated endothelial injury rather than being attributed solely to GVHD. This distinction is particularly important in patients with concurrent GVHD and TA-TMA, where elevated ST2 may reflect overlapping epithelial and vascular injury. Recognition of the endothelial component may prompt evaluation for concomitant TA-TMA and facilitate mechanism-directed therapy rather than escalation of immunosuppression alone.

Recent prospective adult studies further support interpreting ST2 as a marker of endothelial vulnerability rather than exclusively a biomarker of GVHD. These findings reinforce the concept that endothelial injury and alloimmune activation represent overlapping but biologically distinct processes, and that elevated ST2 should prompt evaluation for concurrent endothelial complications, including TA-TMA, rather than automatically leading to escalation of immunosuppression (43).

Renal biomarkers provide an equally important window into systemic endothelial injury (46). Among these, the random urine protein-to-creatinine ratio (rUPCR) has emerged as one of the most clinically valuable biomarkers in pediatric HSCT because it is inexpensive, widely available, rapidly obtained, and easily incorporated into routine transplant monitoring (47). Beyond its established role in TA-TMA diagnosis, longitudinal monitoring of proteinuria provides continuous assessment of renal endothelial injury, correlates with disease severity, and complements markers of systemic complement activation. Importantly, persistent moderate-to-severe proteinuria is uncommon in pediatric HSCT recipients in the absence of endothelial injury. Longitudinal analyses demonstrate that when significant proteinuria occurs without TA-TMA, an alternative major clinical event is typically identifiable, such as sepsis, high urine protein because of hematuria with cystitis, sinusoidal obstruction syndrome, or pre-existing chronic kidney disease. These observations support proteinuria as a robust marker of endothelial injury rather than a nonspecific indicator of kidney dysfunction and reinforce its value as a practical biomarker for routine longitudinal monitoring after HSCT (24). Because TA-TMA is a multisystem endothelial disorder, longitudinal assessment of other organ involvement remains an essential component of treatment monitoring. The major organ manifestations and practical assessment of organ recovery are summarized in **Table 3**.

**Table 3:** Practical organ manifestations of TA-TMA and assessment of organ recovery.

Organ system	Clinically significant TA-TMA involvement	Practical assessment of response/recovery
Kidney	Proteinuria (rUPCR ≥1 mg/mg), acute kidney injury, declining kidney function, dialysis requirement.	Improvement or resolution of proteinuria (rUPCR<1mg/mg), recovery of kidney function toward baseline, dialysis independence when applicable.
Pulmonary	Pulmonary hypertension, oxygen requirement, respiratory failure, mechanical ventilation.	Resolution of pulmonary hypertension, decreasing oxygen requirement, liberation from ventilatory support.
Cardiovascular	Ventricular dysfunction, pulmonary hypertension, pericardial effusion, hemodynamic compromise.	Recovery of cardiac function and resolution of effusions and hemodynamic abnormalities.
Central nervous system	PRES, seizures, encephalopathy, focal neurologic deficits, CNS hemorrhage.	Resolution or marked improvement of neurologic manifestations without new events.
Gastrointestinal	Intestinal TA-TMA, severe abdominal pain, GI bleeding, ileus, bowel ischemia or strictures.	Resolution of GI bleeding and abdominal symptoms with recovery of intestinal function.
Serosal/endothelial injury	Pleural effusions, ascites, pericardial effusions attributable to endothelial injury.	Resolution of effusions without ongoing drainage or intervention.
Overall organ response	One or more organs affected by TA-TMA.	Complete response: recovery of all affected organs. Clinically meaningful partial response: objective improvement in all affected organs without complete recovery. No response: persistent or progressive dysfunction in any involved organ.

This table provides a concise clinical summary of the major organ manifestations of transplant-associated thrombotic microangiopathy (TA-TMA) and practical assessment of organ recovery during therapy. It complements the International Consensus diagnostic and response criteria.

CNS, central nervous system; GI, gastrointestinal; PRES, posterior reversible encephalopathy syndrome; rUPCR, random urine protein-to-creatinine ratio; TA-TMA, transplant-associated thrombotic microangiopathy.

This condensed table is intended as a practical clinical reference. Detailed quantitative organ-specific response definitions are available in the International Consensus Response Criteria.

In addition to circulating biomarkers, composite clinical indices have emerged as practical tools for assessing endothelial vulnerability. The Endothelial Activation and Stress Index (EASIX) provides a practical, readily available index of endothelial stress and has shown utility in adult HSCT cohorts. However, its performance has been limited in pediatric complement-mediated TA-TMA (40, 48).

### 4.3 Inflammatory biomarkers and the interferon response

Increasing evidence suggests that inflammatory pathways interact closely with complement activation during TA-TMA. Among these, CXCL9 has emerged as a promising biomarker reflecting interferon-γ activity and persistent immune activation. Longitudinal monitoring demonstrates that CXCL9 trajectories provide information complementary to complement biomarkers, identifying patients in whom inflammatory signaling remains active despite pharmacodynamic complement control. These observations support the concept that endothelial recovery is influenced by both complement-mediated injury and concurrent inflammatory processes, providing a biological rationale for integrating inflammatory biomarkers into precision therapeutic monitoring (49–52).

### 4.4 Integrating biomarkers into clinical practice

No single biomarker fully captures the complex biology of TA-TMA. Instead, optimal assessment requires integration of complementary biomarkers reflecting distinct biological processes, including systemic terminal complement activation (sC5b-9), endothelial injury (ST2, EASIX), organ-specific vascular injury (rUPCR), inflammatory activation (CXCL9), and pharmacodynamic measures of complement inhibition (e.g., CH50, drug levels). Interpreted longitudinally rather than as isolated measurements, these biomarkers provide a multidimensional assessment of disease activity, therapeutic response, and endothelial recovery.

Importantly, biomarkers should be viewed according to the biological process they represent rather than as independent laboratory tests. This biological framework allows clinicians to distinguish persistent complement activation from ongoing endothelial injury or residual inflammatory signaling, thereby improving interpretation of treatment response and recovery.

Although the clinical availability of individual biomarkers currently varies across transplant centers, laboratory accessibility should not determine their clinical importance. The primary goal of biomarker discovery is to identify the biological pathways that are most informative for diagnosis, risk stratification, and therapeutic monitoring. Once clinical utility has been established, broader assay standardization and implementation become technical rather than scientific challenges. This evolution has occurred repeatedly throughout transplantation and laboratory medicine. Ultimately, precision complement therapeutics should be guided by biological relevance rather than the current availability of individual assays. As clinical testing continues to expand, integrating complementary biomarkers into routine transplant practice has the potential to further individualize therapy and improve patient outcomes.

The growing body of prospective pediatric and adult studies also demonstrates that biomarker performance is influenced by patient population, transplant characteristics, and disease biology. Accordingly, biomarkers should not be viewed as universally interchangeable diagnostic tests but rather as biological tools whose interpretation depends on the clinical context in which they are applied.

## 5 Beyond TA-TMA: translating precision complement therapeutics across transplantation

The lessons learned from TA-TMA extend well beyond the management of a single transplant complication. By establishing endothelial injury and complement activation as clinically actionable therapeutic targets, TA-TMA has fundamentally changed how transplant physicians view a broad spectrum of post-transplant complications. Rather than representing isolated disease entities, many transplant-associated syndromes are now recognized to share overlapping mechanisms of endothelial dysfunction, inflammation, coagulation, and complement activation. Although the relative contribution of these pathways varies among individual disorders, the conceptual framework established through TA-TMA has provided a foundation for mechanism-based investigation across transplantation.

### 5.1 Endothelial injury as a common biological pathway

One of the most important conceptual advances arising from TA-TMA research has been the recognition that endothelial injury is a central biological process underlying multiple transplant complications. Conditioning-related toxicity, calcineurin inhibitors, infections, graft-versus-host disease, and inflammatory cytokines all converge on the vascular endothelium, initiating a cascade of endothelial activation, complement amplification, coagulation abnormalities, and tissue ischemia. TA-TMA represents one manifestation of this process, but similar endothelial biology contributes to the pathogenesis of numerous post-transplant complications. This evolving understanding has shifted the focus from treating individual syndromes in isolation toward identifying shared biological mechanisms that may represent common therapeutic targets.

### 5.2 Shared mechanisms across transplant complications

Increasing evidence supports a role for endothelial injury and complement activation in several severe transplant complications beyond classical systemic TA-TMA. Some evidence suggests that complement activation may contribute to the pathobiology of acute and chronic GVHD, although its precise role remains incompletely defined. Prospective studies have associated systemic complement activation with adverse transplant outcomes, and early clinical trials of complement inhibition in acute GVHD have demonstrated biological activity. Gastrointestinal TA-TMA frequently coexists with acute GVHD, where microvascular injury contributes to severe gastrointestinal bleeding, abdominal pain, steroid refractoriness, and poor clinical outcomes. At the tissue level, however, deposition of terminal complement components (C5b-9) and MASP-2 appears characteristic of TA-TMA rather than GVHD, providing a potential means of distinguishing complement-mediated microangiopathy from acute GVHD in patients with overlapping clinical manifestations. Together, these findings support continued investigation of complement as a shared but disease-specific therapeutic pathway across post-transplant endothelial complications (21, 43, 53–58).

Endothelial dysfunction has been implicated in diffuse alveolar hemorrhage, sinusoidal obstruction syndrome, capillary leak syndromes, chronic kidney injury after transplantation and other complications. While complement activation is unlikely to be the dominant driver in all of these conditions, recognition of shared vascular biology has stimulated investigation into disease-specific contributions of endothelial injury and complement dysregulation.

Importantly, these observations should not be interpreted as evidence that all endothelial complications require complement inhibition. Rather, they emphasize the importance of identifying the biological pathways that predominate within each clinical syndrome and selecting therapies accordingly.

### 5.3 From syndrome-based to mechanism-based medicine

Perhaps the most enduring contribution of TA-TMA has been the transition from syndrome-based to mechanism-based management of transplant complications. Historically, post-transplant syndromes were classified according to their predominant clinical manifestations or affected organs. Increasing integration of biomarkers, histopathology, PK/PD monitoring, and mechanistic studies now allows clinicians to identify the underlying biological processes driving disease in individual patients.

This approach has important implications for future clinical trials. Rather than evaluating therapies solely within traditional disease categories, emerging studies may increasingly enroll patients based on shared biological features such as complement activation, endothelial dysfunction, or persistent inflammatory signaling. Such biomarker-informed trial designs have the potential to improve patient selection and accelerate development of precision therapeutics across transplantation.

## 6 Future directions for personalized complement therapy

The management of TA-TMA has progressed from empirical supportive care to mechanism-directed complement inhibition guided by PK/PD monitoring and longitudinal biomarker assessment. This evolution provides a framework for the next generation of personalized complement therapeutics in transplantation (**Figure 4**). Future advances will likely extend beyond development of additional complement inhibitors to include improved patient selection, earlier identification of disease, individualized therapeutic strategies, and combination approaches targeting complementary biological pathways.

**Figure 4:**
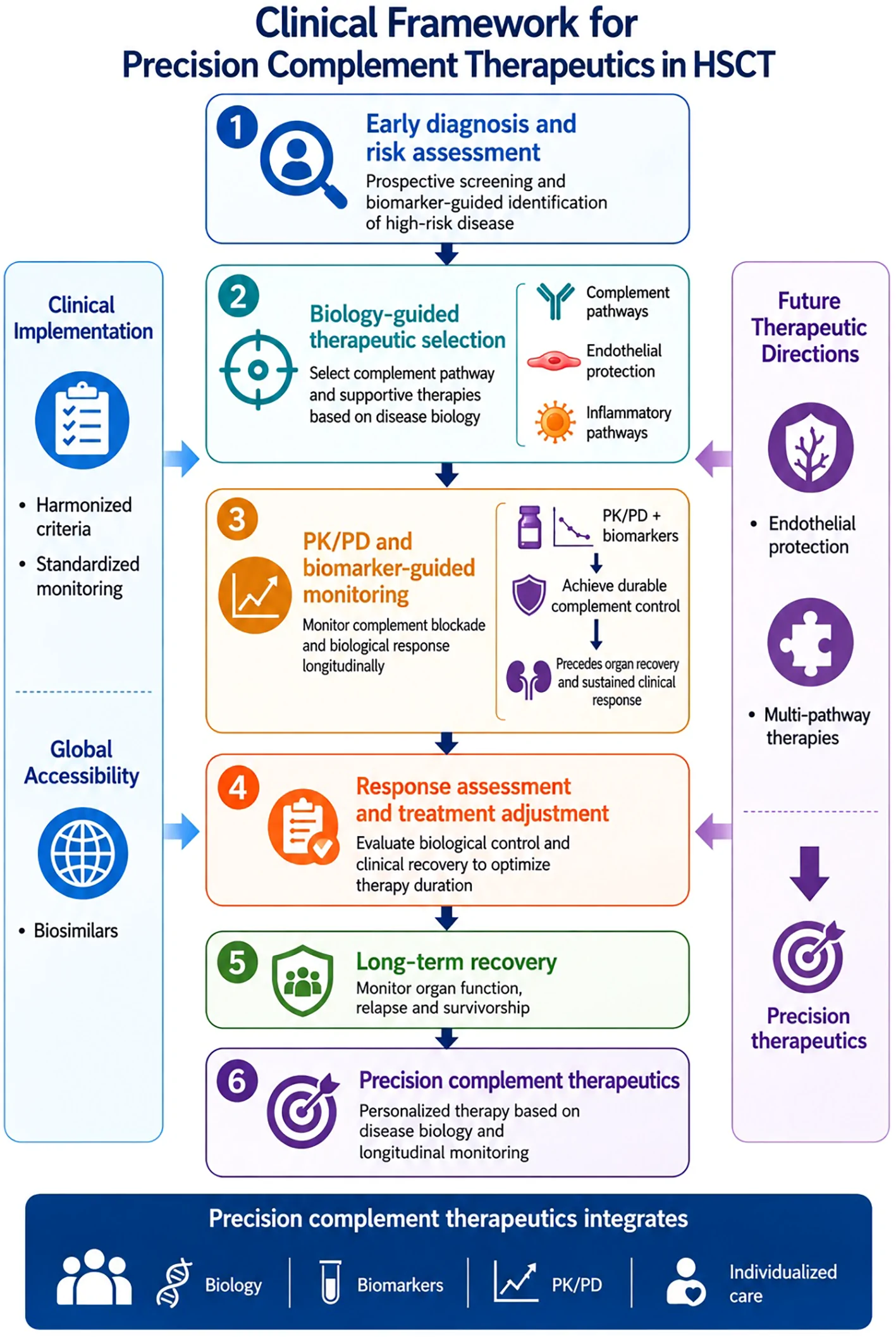
Clinical framework for precision complement therapeutics in HSCT. Proposed approach integrating early diagnosis, biology-guided therapeutic selection, PK/PD and biomarker monitoring, response assessment, and long-term recovery. Harmonized clinical practices, improved therapeutic accessibility, and emerging multipathway strategies support individualized treatment.

### 6.1 Earlier identification of high-risk patients

One of the major goals of future research is to identify patients with evolving endothelial injury before irreversible organ damage develops. Longitudinal studies demonstrate that biological changes frequently precede overt clinical manifestations of TA-TMA, creating opportunities for earlier intervention (18, 59). Integration of complement activation markers with biomarkers of endothelial injury, inflammation, and organ dysfunction may allow identification of high-risk patients before the development of established microangiopathy. Future predictive models will likely combine serial biomarker trajectories with clinical variables to support individualized risk assessment and guide timing of therapeutic intervention.

### 6.2 Expanding precision complement therapeutics

The therapeutic landscape is rapidly expanding beyond eculizumab (13). Multiple complement inhibitors targeting different components of the classical, lectin, alternative, and terminal complement pathways are now entering clinical practice or active investigation. Rather than viewing these agents as interchangeable, future therapeutic strategies should seek to match the dominant biological mechanism to the most appropriate therapeutic target. Such an approach may allow individualized selection of complement inhibitors based on disease biology, complement activation profile, and clinical phenotype rather than adopting a uniform treatment strategy for all patients.

### 6.3 Harmonization and broader implementation of precision complement therapeutics

As precision complement therapeutics continue to evolve, maximizing their clinical impact will require not only the development of new therapies but also broader implementation of evidence-based management strategies. International consensus diagnostic criteria have substantially improved recognition of TA-TMA, yet opportunities remain to further harmonize response assessment, biomarker monitoring, PK/PD-guided dosing strategies, and clinical endpoints used in both routine practice and clinical trials (33, 47, 60). Greater standardization would facilitate comparison across studies, reduce variability in patient management, and accelerate multicenter collaboration.

The recent FDA approval of the MASP-2 inhibitor narsoplimab represents a major milestone in TA-TMA by establishing the first therapy specifically approved for this disease and expanding complement-targeted treatment beyond terminal C5 blockade to the lectin pathway. Clinical studies have demonstrated encouraging response rates of approximately 60% for variable TA-TMA severity together with improved survival, and experience from expanded-access programs suggests activity across both adult and pediatric populations, including some patients previously treated with C5 inhibitors (61–63). The availability of a pathway-specific complement inhibitor represents a major advance toward individualized complement therapeutics and is expected to facilitate broader clinical adoption of complement-targeted therapy in TA-TMA. Regulatory perspectives currently differ internationally; while the FDA approved narsoplimab, the EMA issued a negative opinion in 2026 because of uncertainty regarding efficacy based primarily on single-arm studies with external comparator data. Important questions nevertheless remain regarding identification of patients most likely to benefit from lectin pathway inhibition, development of biomarkers for treatment selection and monitoring, and the optimal integration of MASP-2 inhibition with terminal complement blockade.

The expanding availability of eculizumab biosimilars represents another important opportunity to improve access to life-saving therapies. By reducing treatment costs, biosimilars may enable earlier intervention and broader implementation of complement inhibition in transplant centers where cost has been a major barrier. Importantly, biosimilars can be integrated into the existing precision medicine framework established for eculizumab, including PK/PD-guided dosing, pharmacodynamic monitoring, and longitudinal biomarker assessment, allowing more rapid adoption into clinical practice while maintaining individualized treatment strategies.

Together, harmonized clinical practice and improved therapeutic accessibility have the potential to extend the benefits of precision complement therapeutics to a larger population of transplant recipients while supporting more consistent evaluation of emerging therapies in future clinical trials.

### 6.4 Multimodal strategies to protect the endothelium

Complement inhibition has transformed the management of TA-TMA but addresses only one component of the complex biological processes driving endothelial injury after HSCT. Future therapeutic strategies will likely combine precision complement inhibition with interventions that reduce endothelial vulnerability throughout the transplant course.

Growing evidence suggests that optimization of supportive care may improve endothelial resilience and reduce the severity of transplant-associated vascular injury. Correction of nutritional deficiencies important for endothelial homeostasis, including vitamins C and D, and emerging data supporting vitamin A supplementation in selected pediatric HSCT populations, represent examples of readily implementable supportive strategies. Likewise, prophylactic administration of N-acetylcysteine has shown promise in reducing endothelial injury and the incidence of TA-TMA, highlighting the potential role of antioxidant approaches in vascular protection (15, 64–69).

Modification of transplant practices may provide additional opportunities to reduce endothelial stress. Improvements in GVHD prophylaxis, including abatacept-based approaches, have been associated with lower rates of severe endothelial complications, suggesting that attenuation of early alloimmune injury may indirectly protect the vascular endothelium. Optimization of infection prevention, careful management of calcineurin inhibitor exposure, and reduction of other endothelial stressors may further complement targeted therapies (21, 70–72).

Finally, increasing understanding of the interactions between complement activation, inflammatory pathways, and endothelial dysfunction provides a rationale for combination biological therapies. Agents targeting pathways such as interferon-γ may prove particularly valuable in selected patients with persistent inflammatory activation, although prospective studies are needed to define the optimal combinations and timing of therapy.

Ultimately, the future of TA-TMA management is likely to extend beyond complement inhibition alone toward integrated strategies that combine endothelial-protective supportive care, optimized transplant practices, targeted immunomodulation, and precision complement therapeutics tailored to the dominant biological processes driving disease in each patient.

## 7 Conclusions

Over the past two decades, TA-TMA has evolved from a poorly understood and frequently fatal transplant complication into one of the most informative clinical models for precision complement therapeutics. Advances in complement biology, prospective clinical studies, PK/PD-guided dosing, and longitudinal biomarker monitoring have transformed the management of TA-TMA from empirical treatment toward individualized, mechanism-based therapy. These advances have substantially improved patient outcomes while establishing principles that extend beyond a single disease.

The continued evolution of the field will depend not only on the development of new complement inhibitors but also on earlier identification of high-risk patients, harmonized diagnostic and response assessment criteria, broader implementation of precision monitoring strategies, and improved global access through biosimilars. At the same time, increasing recognition that endothelial injury results from complex interactions among complement activation, inflammation, immune dysregulation, and transplant-related stressors supports future therapeutic approaches that integrate complement inhibition with endothelial-protective supportive care and targeted immunomodulation.

Ultimately, the greatest legacy of TA-TMA may be its role in demonstrating that understanding disease biology can fundamentally transform clinical practice. The principles established through the study of TA-TMA, including longitudinal biological monitoring, PK/PD-guided therapy, complementary biomarker integration, and individualized treatment strategies, provide a framework for the future of precision therapeutics across transplantation. As our understanding of endothelial biology continues to expand, TA-TMA will likely remain a model for developing personalized approaches to vascular complications throughout HSCT and, potentially, transplantation more broadly.
